# Effect of Diosgenin on the Circulating MicroRNA Profile of Ovariectomized Rats

**DOI:** 10.3389/fphar.2020.00207

**Published:** 2020-03-06

**Authors:** Zhiguo Zhang, Lihua Xiang, Yuhan Wang, Yanhua Jiang, Yin Cheng, Gary Guishan Xiao, Dahong Ju, Yanjing Chen

**Affiliations:** ^1^Institute of Basic Theory, China Academy of Chinese Medical Sciences, Beijing, China; ^2^School of Pharmaceutical Science, Dalian University of Technology, Dalian, China; ^3^Functional Genomics and Proteomics Laboratory, Osteoporosis Research Center, Creighton University School of Medicine, Omaha, NE, United States; ^4^Experimental Research Center, China Academy of Chinese Medical Sciences, Beijing, China

**Keywords:** diosgenin, circulating microRNAs, ovariectomized rat, osteoclastogenesis, tibia

## Abstract

The present study aimed to assess the changes in circulating microRNA (miRNA) expression profiles associated with the potential osteoprotective effect of diosgenin (DIO) in ovariectomized (OVX) rats. Wistar rats (female) were subjected to a sham operation (SHAM group) or ovariectomy. OVX rats were treated with DIO (DIO group) or vehicle (OVX group) for 12 weeks. Following treatment, the serum estradiol, bone turnover biomarker levels, and the microarchitecture of tibias were assayed. Based on miRNA microarray and qRT-PCR analyses, differentially expressed (DE) circulating miRNAs were identified between the OVX and SHAM groups (comparison A) and between the DIO and OVX groups (comparison B). Furthermore, putative target genes of shared DE miRNAs with opposite expression trends in the two comparisons were predicted by ingenuity pathway analysis (IPA). Finally, the expression levels of the putative target genes in serum and tibia were validated by qRT-PCR. The micro-CT results demonstrated that DIO had a substantial anti-osteopenic effect on the tibias of OVX rats. In total, we found 5 DE circulating miRNAs (four upregulated and one downregulated) in comparison A and 21 DE circulating miRNAs (15 upregulated and 6 downregulated) in comparison B. However, only one DE circulating miRNA (rno-miR-20a-5p) had opposite expression trends between the two comparisons. Including rno-miR-20a-5p, 7 of the 10 selected DE circulating miRNAs between the two comparisons passed qRT-PCR validation. Specifically, based on qRT-PCR validation, DIO upregulated the expression of rno-miR-20a-5p and downregulated that of three target genes (*Tnf*, *Creb1*, and *Tgfbr2*) of the “osteoclast differentiation” pathway in the tibias of OVX rats. Our results suggested that DIO could change the circulating miRNA profile of OVX rats and inhibited the downregulation of miR-20a-5p in serum and tibia. DIO might exert an anti-osteoclastogenic effect on OVX rats by upregulating the expression of miR-20a-5p in circulation and bone tissue.

## Introduction

In the last two decades years, lots of biomarkers of bone metabolism have been found with increased sensitivity and specificity. For osteoporosis, some academic organizations have recommended the crosslinked C-terminal telopeptide (CTX) and type I collagen *N*-propeptide (PINP) as biomarkers of bone resorption and formation. However, these biomarkers are lack of specificity for bone tissue. As a result, researchers switch their attention to microRNAs (miRNAs).

As a kind of small, non-coding RNAs, miRNAs have been shown to be post-transcriptional regulators of gene expression. Undoubtedly, miRNAs also play a part in normal bone metabolism and osteopathy including osteoporosis (Gennari et al., 2017). Recently, some circulating miRNAs, which have been identified as blood-based biomarkers for the diagnosis of osteoporosis in humans (Cao et al., 2014; Li et al., 2014) or animal models (Chen et al., 2016), have drawn increasing attention. For example, serum level of miRNA-214 was thought to be correlated with bone formation of old fracture patients negatively (Wang et al., 2013). Further study found that ATF4 is the target mRNA of miRNA-214, which indirectly regulates expression of osteogenic transcription factor in osteoblasts during cell differentiation (Yang et al., 2004).

Diosgenin (DIO), an aglycone of the steroid saponin, have been proven to be a phytoestrogen (Au et al., 2004; Jesus et al., 2016), but the estrogen-like effect of DIO is milder than real estrogen (Chiang et al., 2011; Zhang et al., 2014; Folwarczna et al., 2016). DIO has an anti-osteopenic effect on the osteoporosis rat model via attenuating of the RANKL/OPG ratio (Zhang et al., 2014) or regulating long non-coding RNAs (Zhang et al., 2018) in bone tissue, but the effect of DIO on the serum miRNA profile of osteoporosis rats induced by ovariectomy remains unknown.

The specific circulating miRNA signatures can be used as potential diagnostic biomarkers, but the question remains whether these biomarkers may represent new drug targets for osteoporosis. In the present study, we explored the effect of DIO on circulating miRNAs in ovariectomized (OVX) rats for the first time. We attempted to answer two questions. First, whether DIO could mitigate the alteration of the serum miRNA profile in OVX rats. Second, if there are suitable reference miRNAs that have the potential to be used as therapeutic biomarkers to evaluate the degree of bone loss.

## Materials and Methods

### Animal Grouping and Treatments

Thirty-six female Wistar rats (6 months old, 300 ± 20.0 g) were purchased from the National Institutes for Food and Drug Control of China (Beijing, China). This animal experiment was approved by the Institutional Ethics Committee of the Institute of Basic Theory, China Academy of Chinese Medical Sciences [SCXK-(Jing) 2017-0005, Beijing, China]. All acclimatized rats underwent either bilateral ovariectomy (*n* = 24) or sham operation (SHAM, *n* = 12). Thereafter, the OVX rats were randomly divided into two groups: the OVX group (OVX, *n* = 12) and the DIO group (DIO, *n* = 12). The DIO group rats were treated with DIO (Sigma-Aldrich, Saint Louis, MO, United States, purity > 95%) prepared as a suspension and administered at 100 mg/kg body weight/day by oral gavage. We select this dose of DIO according to previous publications from us (Zhang et al., 2018) and other researchers (Gong et al., 2010, 2011). The OVX and SHAM group rats were administered distilled water by oral gavage. During the course of the experiments, all of the rats were subjected to a 12 h:12 h light/dark cycle and fed standard chow. At 1 week after the operation, all of the acclimatized rats started to undergo 12-week treatments. No animal died in the treatment period.

### Preparation of Specimens

After the last drug administration, the animals were anesthetized by intraperitoneal injection of xylazine (12 mg/kg body weight) and ketamine (80 mg/kg body weight) and sacrificed by exsanguination. The abdominal aorta was punctured prior to death to collect blood specimens into tubes. Thereafter, the blood samples were centrifuged at 3,000 × *g* (4°C, 10 min) and further aliquoted and stored at −80°C until use. The left tibias were dissected and stored at −80°C for microarray and quantitative real-time reverse transcription polymerase chain reaction (qRT-PCR) assays. The right tibias were dissected and stored at −20°C for measurements of microarchitecture by microcomputerized tomography (micro-CT).

### Assay of Level of Serum Estradiol and Bone Turnover Biomarkers

The serous level of estradiol, tartrate-resistant acid phosphatase (TRAP), and alkaline phosphatase (ALP) in rats was determined by an electrochemiluminescence immunoassay (ECLIA) Kit (Roche Diagnostics, Mannheim, Germany) and enzyme-linked immunosorbent assays (ELISA) Kit (Sunbio, Inc., Beijing, China) following the manufacturer’s protocol, respectively.

### Micro-CT Scanning of Tibias

The right tibias of rats were scanned by SkyScan 1174 micro-CT (SkyScan, Antwerp, Belgium). The volume of interest (VOI) was measured starting from the lowest point of the growth plate and extended toward the diaphysis for 1.5 mm. The acquisition conditions of the data were set as voltage (50 kV), current (800 mA), 0.5-mm-thick filter (aluminum), 12 μm/pixel size, 5300 ms exposure time, and 0.8°/step wise rotation. The reconstruction and analysis of VOI data were performed using CTanalyzer, an embedded software. The parameters evaluated in the microarchitecture of trabeculae included bone mineral density (BMD), trabecular bone volume (BV/TV), trabecular separation (Tb.Sp), trabecular number (Tb.N), trabecular thickness (Tb.Th), degree of anisotropy (DA), and structure model index (SMI).

### miRNA Microarray Assay

KangChen Bio-tech Company (Shanghai, China) conducted the miRNA microarray assay. Total RNA from 400 μL of serum from SHAM, OVX, or DIO group rats was harvested using the miRNeasy Mini Kit (Qiagen, Valencia, CA, United States) and TRI Reagent BD kit (Molecular Research Center, Cincinnati, OH, United States) in accordance with the provided protocol. After RNA quantity passed the test, the samples were labeled using the Hy3/Hy5 Power labeling kit (Exiqon, Vedbaek, Denmark) and hybridized on the LNA Array (Exiqon, Vedbaek, Denmark). The miRNA array covered all 3100 miRNAs of *Homo sapiens*, *Mus musculus*, and *Rattus norvegicus* from the miRBase 18.0 database.

After washing, the chips were scanned with the Axon GenePix 4000B scanner (Molecular Devices, Sunnyvale, CA, United States). Scanned images were then imported into GenePix Pro 6.0 software (Molecular Devices, Sunnyvale, CA, United States) for data extraction and analyses. We set a threshold (fold change ≥ 2 and *P*-value < 0.05) for screening differentially expressed (DE) miRNAs in serum between OVX and SHAM group rats (comparison A) and in serum between DIO and OVX group rats (comparison B), respectively.

### Target Gene Prediction and Analyses

Identical DE miRNAs with opposite expression trends between comparison A and comparison B were imported into ingenuity pathway analysis (IPA). We predicted putative targets of the miRNAs by setting confidence to “experimentally observed” based on an integrated database that included Ingenuity^®^ Knowledge Base, TarBase, TargetScan, and miRecords. Thereafter, identified mRNA targets were subjected to Gene Ontology (GO) and KEGG pathway enrichment analysis using DAVID (Huang da et al., 2009).

### Validation of Genes by qRT-PCR

Gene quantitative analysis was performed with qRT-PCR by using SYBR RT-PCR kits (Takara, Dalian, China) and the ABI 7500 system (Applied Biosystems, Foster City, CA, United States). Using the 2^–ΔΔCt^ cycle threshold method, the expression of each miRNA or mRNA in tibia bone was represented by the relative ratio to the expression of the internal control gene U6 or *Gapdh*, respectively. For quantitative detection of the expression level of each miRNA in serum samples, single-stranded cel-miR-39 (25 fmol, Invitrogen, Carlsbad, CA, United States) as an internal control gene was spiked into 400 μL of serum. All primers used for detecting miRNAs or target mRNAs are listed in Tables 1 or 2, respectively.

**TABLE 1:** Primers of miRNAs.

**Name**	**Primers**
cel-miR-39	F: 5′-UCACCGGGUGUAAAU CAGCUUG-3′ R: 5′-TCACCGGGTGTAAAT CAGCTTG-3′
U6	F: 5′-GCTTCGGCAGCACATATACTAAAAT-3′ R: 5′-CGCTTCACGAATTTGCGTGTCAT-3′
rno-miR-465-5p	GSP: 5′-ACGGTGCTGGTGTGGT-3′ R: 5′-CAGTGCGTGTCGTGGAGT-3′
rno-miR-344a-3p	GSP: 5′-CTGTGTCGTATCCAGTGCA-3′ R: 5′-CAGTGCGTGTCGTGGAGT-3′
rno-miR-539-3p	GSP: 5′-TTCTTTTCGTCGTATCCAGTGC-3′ R: 5′-CAGTGCGTGTCGTGGAGT-3′
rno-miR-345-5p	GSP: 5′-CCCCTAGTCCAGTGCGTC-3′ R: 5′-CAGTGCGTGTCGTGGAGT-3′
rno-miR-20a-5p	GSP: 5′-GTGCAGGTAGGTCGTATCCA-3′ R: 5′-CAGTGCGTGTCGTGGAGT-3′
rno-miR-10a-5p	GSP: 5′-AGATCCGAATTTGTGGTCGT-3′ R: 5′-CAGTGCGTGTCGTGGAGT-3′
rno-miR-32-5p	GSP: 5′-AAGTTGCAGTCGTATCCAGTG-3′ R: 5′-CAGTGCGTGTCGTGGAGT-3′
rno-miR-126a-3p	GSP: 5′-TGCGGTCGTATCCAGTGC-3′ R: 5′-CAGTGCGTGTCGTGGAGT-3′
rno-miR-433-5p	GSP: 5′-TGAGCCTGTCATTATTCGTCG-3′ R: 5′-CAGTGCGTGTCGTGGAGT-3′
rno-miR-540-5p	GSP: 5′-TCACCCTCTGACTCTGTGT-3′ R: 5′-CAGTGCGTGTCGTGGAGT-3′
	

**TABLE 2:** Primers of mRNA targets.

**Name**	**Primers**
*Gapdh*	F: 5′-GGAAAGCTGTGGCGTGAT-3′ R: 5′-AAGGTGGAAGAATGGGAGTT-3′
*Tnf*	F: 5′-CCCTTTATCGTCTACTCCTCAGA-3′ R: 5′-TGAGCATCGTAGTTGTTGGAAA-3′
*Creb1*	F: 5′-CAAACATACCAGATTCGCACAG-3′ R: 5′-TCTCTTTCGTGCTGCTTCTT-3′
*Tgfbr2*	F: 5′-ATGTCTACTCCATGGCTCTAGT-3′ R: 5′-TCTCTCAGCACGTTGTCTTTC-3′
*Jak1*	F: 5′-CCAAAGCAATTGAGACCGATAAG-3′ R: 5′-CCAGACATCAGAGGCGATATAAA-3′
*Pparg*	F: 5′-AAGTGACTCTGCTCAAGTATGG-3′ R: 5′-ATGAATCCTTGTCCCTCTGATATG-3′

### Statistical Analysis

Mean ± standard deviation was used to present all the values and all statistical analyses were completed by SPSS 19.0 (IBM Corp., Armonk, NY, United States). The normality tests of all data were carried out by the Kolmogorov–Smirnov test method. The least significant difference (LSD) test and one-way analysis of variance (ANOVA) were used to test the differences in evaluated parameters between groups. Statistical significance of the *P*-value was defined as <0.05.

## Results

### Influence of DIO on the Serous Level of Estradiol and Bone Turnover Biomarkers

**FIGURE 1:**
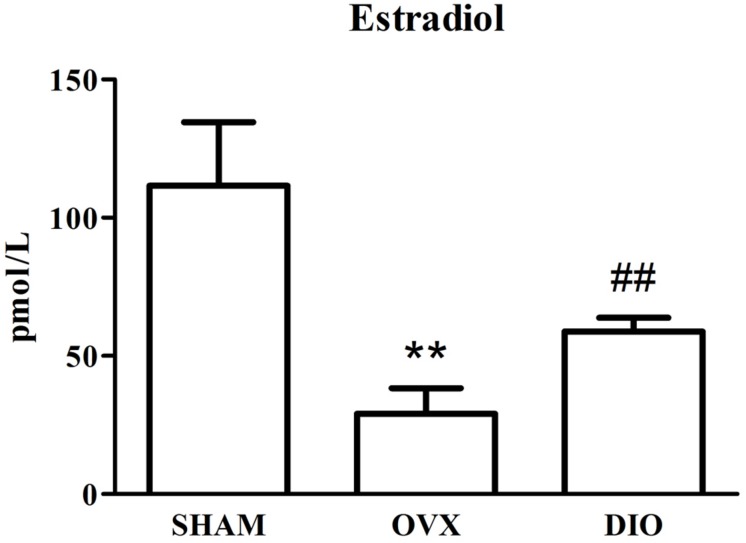
Effect of DIO on level of serous estradiol. **P < 0.01 vs. the SHAM group; ^##^*P* < 0.01 vs. the OVX group.

**FIGURE 2:**
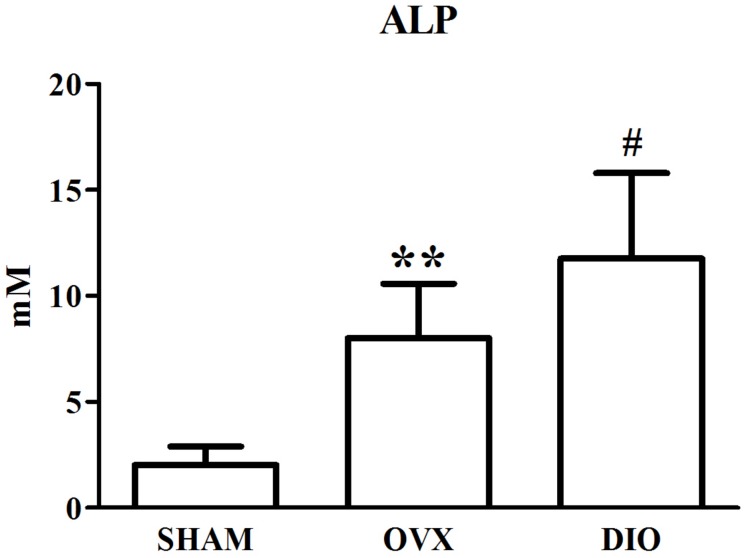
Effect of DIO on serous level of alkaline phosphatase (ALP). **P < 0.01 vs. the SHAM group; ^#^*P* < 0.05 vs. the OVX group.

**FIGURE 3:**
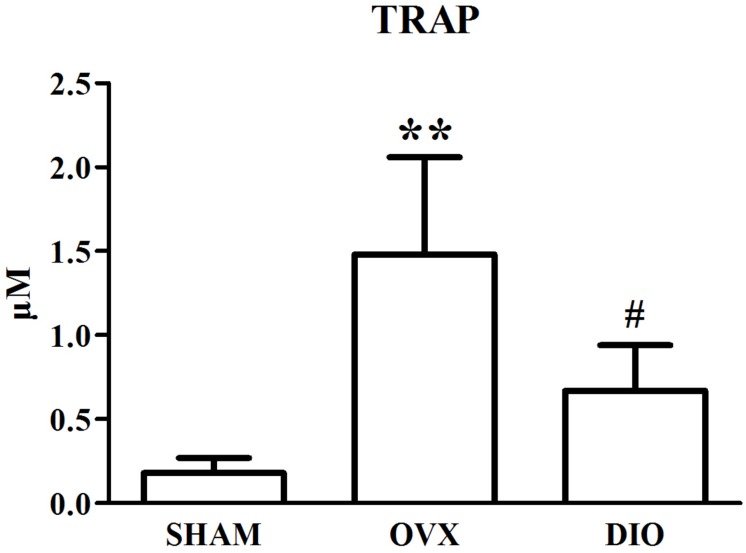
Effect of DIO on serous level of tartrate-resistant acid phosphatase (TRAP). **P < 0.01 vs. the SHAM group; ^#^*P* < 0.05 vs. the OVX group.

Figures 1–3 show the serum concentrations of estradiol, ALP, and TRAP in rats from different groups after 12 weeks of treatment. After treatment, the serum estradiol levels in the OVX group rats were remarkably lower than those in the SHAM rats (*P* < 0.01). However, the serum estradiol levels in the DIO group rats were significantly higher than those in the OVX group rats. The ALP and TRAP levels in OVX rats were significantly higher in comparison to those in SHAM rats (*P* < 0.01). Nevertheless, DIO could increase the level of ALP and decrease the level of TRAP in model rats compared to those in OVX group rats (*P* < 0.05).

### Effect of DIO on BMD and Parameters of Trabecular Bone Microarchitecture

**FIGURE 4:**
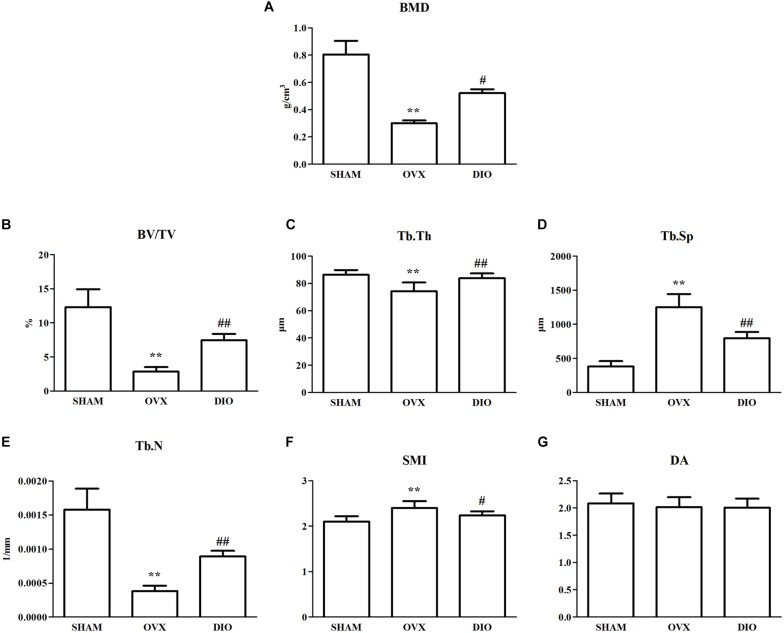
Effect of DIO on bone mineral density and trabecular bone microarchitecture. **(A)** BMD, **(B)** BV/TV, **(C)** Tb.Th, **(D)** Tb.Sp, **(E)** Tb.N, **(F)** SMI, and **(G)** DA. **P < 0.01 vs. the SHAM group; ^##^*P* < 0.01 vs. the OVX group; ^#^*P* < 0.05 vs. the OVX group. BMD, bone mineral density; BV/TV, trabecular bone volume; Tb.Sp, trabecular separation; Tb.N, trabecular number; Tb.Th, trabecular thickness; DA, degree of anisotropy; SMI, structure model index.

**FIGURE 5:**
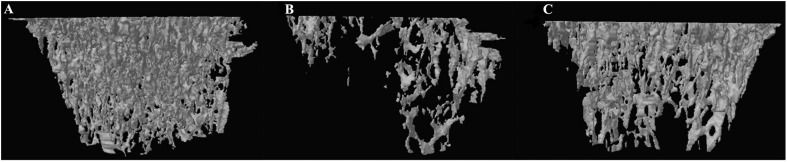
Representative 3-D trabecular bone microarchitecture of tibia starting from the lowest point of growth plate and extended toward the diaphysis for 1.5 mm from each group. **(A)** SHAM, **(B)** OVX, and **(C)** DIO.

The results from micro-CT scanning indicated that BMD, Tb.N, BV/TV, and Tb.Th of the OVX group were significantly decreased (*P* < 0.01), while Tb.Sp and SMI were increased in comparison with those of the SHAM group (*P* < 0.01). To some extent, DIO treatment significantly relieved the deterioration of the trabecular bone microarchitecture induced by ovariectomy (Figures 4A–G, 5A–C).

### Regulation of DIO on the Serum miRNA Expression Profile

The microarray results suggested that the expression of five circulating miRNAs in serum in OVX rats was altered (more than twofold) compared to that in the SHAM group. Specifically, four miRNAs were upregulated, and one miRNA was downregulated (Table 3). However, there were 21 circulating DE miRNAs between the serum from DIO and OVX group rats. Of the 21 DE miRNAs, 15 were upregulated and 6 were downregulated (Table 4). The data in Tables 3, 4 demonstrated that rno-miR-20a-5p, the only one shared DE miRNA with opposite expression trends was identified.

**TABLE 3:** Differentially expressed miRNAs in serum between the OVX group and SHAM group.

**miRNA**	**Fold change**	***P*-value**
**Up-regulated miRNAs**		
rno-miR-539-3p	11.208	0.002
rno-miR-345-5p	3.471	0.013
rno-miR-344a-3p	2.739	0.024
rno-miR-465-5p	2.006	0.021
**Down-regulated miRNA**		
rno-miR-20a-5p	–2.030	0.034
		

**TABLE 4:** Differentially expressed miRNAs in serum between the DIO group and OVX group.

**miRNA**	**Fold change**	***P*-value**
**Up-regulated miRNAs**		
rno-miR-10a-5p	7.567	0.047
rno-miR-126a-3p	3.567	0.004
rno-miR-32-5p	3.074	0.017
rno-miR-742-3p	2.981	0.031
rno-miR-99a-5p	2.898	0.003
rno-miR-702-3p	2.796	0.030
rno-miR-100-5p	2.744	0.024
rno-miR-301a-3p	2.640	0.014
rno-miR-15b-5p	2.564	0.018
rno-let-7i-5p	2.445	0.020
rno-miR-374-5p	2.411	0.010
rno-miR-30b-5p	2.323	0.041
rno-miR-208a-5p	2.313	0.049
rno-miR-148b-3p	2.280	0.034
rno-miR-20a-5p	2.012	0.030
**Down-regulated miRNAs**		
rno-miR-540-5p	–3.120	0.026
rno-miR-433-5p	–2.849	0.027
rno-miR-30c-1-3p	–2.664	0.003
rno-miR-185-3p	–2.248	0.022
rno-miR-465-5p	–2.143	0.010
rno-miR-98-5p	–2.002	0.025

### Target Gene Prediction and Analyses

**FIGURE 6:**
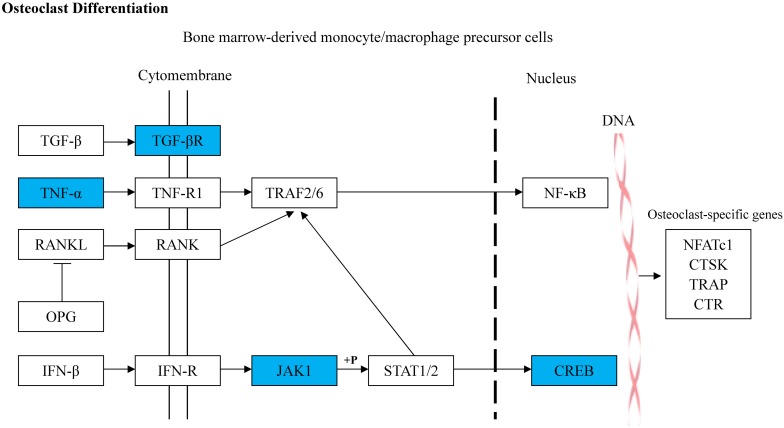
Diagram illustrating the predicted target genes of rno-miR-20a-5p in osteoclast differentiation pathway based on KEGG. The blue colored genes represented the validated downregulated target genes of rno-miR-20a-5p. The white colored genes were the related genes introduced into this pathway. The arrow lines represent the relationship between upstream molecules and downstream molecules.

rno-miR-20a-5p was imported into IPA, and 46 target genes were predicted (Supplementary Table S1). GO and KEGG pathway analyses of the 46 target genes were performed in DAVID. The top five KEGG pathways associated with miRNA or bone metabolism are listed in Table 5. Among the top five pathways listed in Table 5, only one pathway, “osteoclast differentiation,” was involved in bone metabolism (Figure 6).

**TABLE 5:** Top five KEGG pathways associated with miRNA and bone metabolism on target genes.

**Pathway**	**P-value**
MicroRNAs in cancer	9.77 × 10^–14^
FoxO signaling pathway	7.92 × 10^–7^
Cell cycle	8.86 × 10^–6^
PI3K-Akt signaling pathway	3.91 × 10^–5^
Osteoclast differentiation	1.60 × 10^–3^

### Validation of miRNAs and Target Genes by qRT-PCR

**FIGURE 7:**
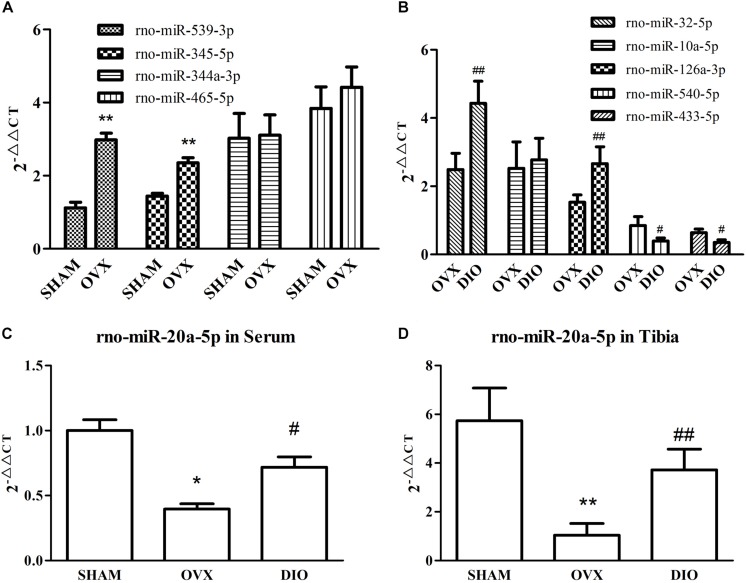
RT-qPCR validation of nine differentially expressed miRNAs from two comparisons. **(A)** The expressions of rno-miR-539-3p, rno-miR-345-5p, rno-miR-344a-3p, and rno-miR-465-5p in serum from SHAM and OVX group rats. **(B)** The expressions of rno-miR-10a-5p, rno-miR-126a-3p, rno-miR-32-5p, rno-miR-540-5p, and rno-miR-433-5p in serum from DIO and OVX group rats. **(C)** The expressions of rno-miR-20a-5p in serum from SHAM, OVX, and DIO group rats. **(D)** The expressions of rno-miR-20a-5p in tibias from SHAM, OVX, and DIO group rats. *P < 0.05 vs. the SHAM group; **P < 0.01 vs. the SHAM group; ^#^*P* < 0.05 vs. the OVX group; ^##^*P* < 0.01 vs. the OVX group.

**FIGURE 8:**
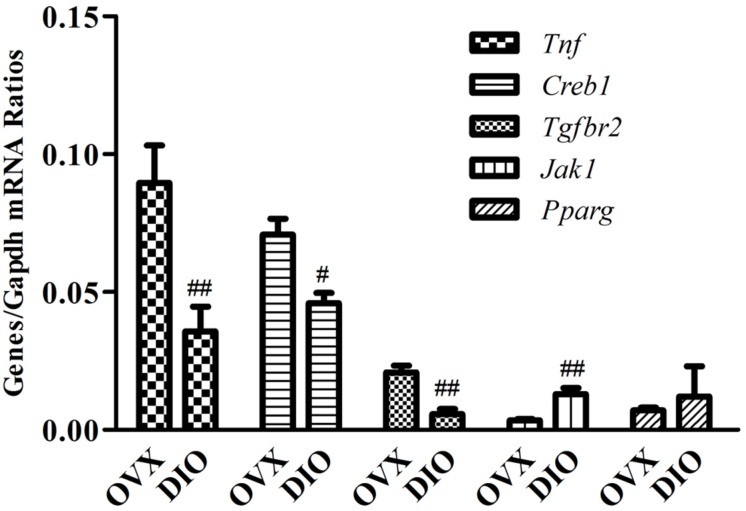
RT-qPCR validation of five predicted target genes (*Creb1*, *Jak1*, *Pparg*, *Tgfbr2*, and *Tnf*) of rno-miR-20a-5p in tibias from OVX and DIO group rats. ^##^*P* < 0.01 vs. the OVX group; ^#^*P* < 0.05 vs. the OVX group.

We assessed the expression of 10 circulating DE miRNAs (rno-miR-465-5p, rno-miR-344a-3p, rno-miR-539-3p, rno-miR-345-5p, rno-miR-20a-5p, rno-miR-10a-5p, rno-miR-32-5p, rno-miR-126a-3p, rno-miR-433-5p, and rno-miR-540-5p) in rats using qRT-PCR. Thereafter, in the tibias of rats, the expression of rno-miR-20a-5p and five of its target genes (*Creb1*, *Jak1*, *Pparg*, *Tgfbr2*, and *Tnf*), which were allocated in the “osteoclast differentiation” pathway, were also validated using qRT-PCR. As a result, the serum miRNA expression results from qRT-PCR were generally consistent with the microarray data except rno-miR-344a-3p, rno-miR-465-5p, and rno-miR-10a-5p (Figure 7). In the tibia of OVX rats, the expression of *Tnf*, *Creb1*, and *Tgfbr2* was downregulated, the expression of *Jak1* was upregulated, and the expression of *Pparg* showed no significant change after 12 weeks of treatment with DIO (Figure 8).

## Discussion

In the present study, for the first time, we explored the action of DIO on the serum circulating miRNA profile of OVX rats.

After ovariectomy, BMD in the tibia of OVX rats was markedly reduced due to a raise in bone turnover in comparison with the rats in the SHAM group. However, DIO treatment increased the BMD of the tibia in comparison with the OVX group. The 12-week DIO treatment significantly increased the level of estradiol and ALP and decreased the level of TRAP (Figures 1–3), indicating that DIO had an estrogen-like effect that could arouse osteoprotective action.

We used micro-CT scanning to determine the osteoprotective action of DIO on OVX rats. A 3-D analysis of bone microarchitecture showed that the tibia bone in the OVX group rats had more bone loss than the DIO-treated rats (Figure 4), which was indicated by the BV/TV, SMI, Tb.N, Tb.Th, and Tb.Sp measurements (Figure 5). The results of micro-CT scanning showed that DIO had a considerable osteoprotective effect on the tibia of rats, similar to previous reports (Chiang et al., 2011; Zhang et al., 2014).

A previous study on the circulating miRNA profile of OVX rats reported changes in the expression of several circulating miRNAs in serum, which may be used as biomarkers of bone loss (Chen et al., 2016), but our present study showed different results. In this study, we found five DE miRNAs in serum between the OVX group and SHAM group (Table 3) and 21 DE miRNAs between the DIO group and OVX group (Table 4). The qRT-PCR assay confirmed the microarray results (Figures 7A,B).

Intriguingly, rno-miR-20a-5p was downregulated in both the serum and tibia of SHAM group rats compared to OVX group rats and was upregulated in DIO group rats (Tables 3, 4). In other words, DIO may exert its anti-osteoporotic effect by regulating the expression of rno-miR-20a-5p in serum or bone tissue. Naturally, our attention focused on rno-miR-20a-5p, and we wondered how rno-miR-20a-5p regulated bone metabolism. First, we validated the expression of rno-miR-20a-5p in serum and tibia bone using qRT-PCR. As expected, the expression of rno-miR-20a-5p in the serum and tibia of DIO group rats shared a similar upregulation effect compared to the OVX group (Figures 7C,D). Second, we analyzed 46 target genes of rno-miR-20a-5p. The results of GO and pathway enrichment analyses showed that the target genes were associated with regulation of transcription and cancer. A line of studies has proven that DIO exerts an antitumor effect by regulating cell transcription (Raju and Mehta, 2009; Chen et al., 2015; Jesus et al., 2016). Notably, among the top 20 pathways, only one pathway, “osteoclast differentiation,” was involved in bone metabolism (Figure 8). Five target genes (*Creb1*, *Jak1*, *Pparg*, *Tgfbr2*, and *Tnf*) were allocated in this pathway, but only three genes (*Tnf*, *Creb1*, and *Tgfbr2*) were validated by qRT-PCR.

miR-20a-5p is a member of the miR-17-92 family and shares common target genes with miR-17-5p. miR-20a-5p/17-5p can bind to *Tnf*, *Creb1*, and *Tgfbr2* mRNA and further downregulate the expression of these mRNAs (Jiang et al., 2011; Li et al., 2015; Fedeli et al., 2016; Jing et al., 2016). TNF-alpha functions in osteoclastogenesis, mostly in synergy with receptor activator of nuclear factor kappa B ligand (RANKL), in promoting pathologic osteoclastogenesis and bone resorption (Azuma et al., 2000; Kitaura et al., 2013; Zhao, 2017). Targeted by the miR-17-92 cluster, TNF-alpha expression was increased in the bone of elderly mice with osteoporosis, while the expression of miR-17/miR-20a-5p decreased (Liu et al., 2015). TGF-beta type II receptor (TβRII) is expressed in osteoclasts and is closely related to the activation of osteoclasts and bone metastasis of tumors. *Tgfbr2* knockout inhibits angiogenesis, tumor cell proliferation, and osteoclastogenesis in metastatic bones (Futakuchi et al., 2009; Meng et al., 2016). *CREB1* is a well-known osteoclastogenic transcription factor, and the differentiation of osteoclast and osteoclastogenic function is regulated by the calcium/calmodulin-dependent protein kinase (CaMK)-CREB pathway (Sato et al., 2006; Yeon et al., 2015). Both macrophages derived from bone marrow and RAW 264.7 cells can express CaMKI and CaMKII gamma isoforms persistently during the differentiation of osteoclasts incubated with RANKL (Ang et al., 2007). qRT-PCR assays validated that the expression of *Tnf*, *Creb1*, and *Tgfbr2* in the tibia was downregulated along with the upregulation of miR-20a-5p in the tibia or serum of DIO-treated rats.

In this study, we found that ovariectomy led to some significant changes in the serum miRNA profile and that the DE miRNAs in serum between OVX and SHAM group rats were not identical to those previously reported (Chen et al., 2016). Treatment with DIO drastically changed the serum miRNA profile of OVX rats but reverse the expression of a single miRNA, miR-20a-5p. The change in circulating miR-20a-5p level can be used to reflect the condition of bone resorption before and after treatment.

## Conclusion

In conclusion, miR-20a-5p might be a target of DIO and associated with the downregulation of osteoclastogenesis. The level of miR-20a-5p in serum to some extent reflected the condition of bone resorption. miR-20a-5p in serum is a potential therapeutic biomarker to evaluate the degree of bone resorption.

## Data Availability Statement

The datasets analyzed for this study can be found in the ArrayExpress (https://www.ebi.ac.uk/arrayexpress/experiments/E-MTAB-8502/).

## Ethics Statement

All of the experiments involving animals were conducted with the approval of the Institutional Ethics Committee of the Institute of Basic Theory, China Academy of Chinese Medical Sciences.

## Author Contributions

ZZ, YJC, GX, and DJ conceived and designed the study. ZZ, LX, YW, YC, and YJ performed the analyses and interpreted the data. ZZ, LX, YJ, YC, and YW collected the data. ZZ, GX, YJC, and DJ drafted and revised the manuscript. All authors read and approved the final manuscript.

## Conflict of Interest

The authors declare that the research was conducted in the absence of any commercial or financial relationships that could be construed as a potential conflict of interest.
