# Supplementary material for: Effect of Diosgenin on the Circulating MicroRNA Profile of Ovariectomized Rats
Source: Front Pharmacol. 2020 Mar 6;11:207. doi: 10.3389/fphar.2020.00207 (PMC7069125; doi:10.3389/fphar.2020.00207)
Supplement: Supplementary file 1 [file Table_1.DOCX]

Supplementary Material

# Supplementary Table

Table S1. Predicted target genes of rno-miR-20a-5p based on IPA.

| miRNA | Source | Confidence | Target genes |
| --- | --- | --- | --- |
| rno-miR-20a-5p | Ingenuity Expert Findings,TargetScan Human,miRecords | Experimentally Observed,Moderate (predicted) | APP |
| rno-miR-20a-5p | TarBase,TargetScan Human,miRecords | Experimentally Observed,High (predicted) | ARID4B |
| rno-miR-20a-5p | Ingenuity Expert Findings,TargetScan Human | Experimentally Observed,High (predicted) | BAMBI |
| rno-miR-20a-5p | miRecords | Experimentally Observed | BCL2 |
| rno-miR-20a-5p | Ingenuity Expert Findings,miRecords | Experimentally Observed | BCL2L11 |
| rno-miR-20a-5p | TargetScan Human,miRecords | Experimentally Observed,High (predicted) | BMPR2 |
| rno-miR-20a-5p | Ingenuity Expert Findings,TargetScan Human | Experimentally Observed,Moderate (predicted) | BNIP2 |
| rno-miR-20a-5p | Ingenuity Expert Findings | Experimentally Observed | CAMTA1 |
| rno-miR-20a-5p | TargetScan Human,miRecords | Experimentally Observed,Moderate (predicted) | CCND1 |
| rno-miR-20a-5p | Ingenuity Expert Findings,TarBase,TargetScan Human,miRecords | Experimentally Observed,High (predicted) | CDKN1A |
| rno-miR-20a-5p | Ingenuity Expert Findings | Experimentally Observed | CREB1 |
| rno-miR-20a-5p | Ingenuity Expert Findings,TargetScan Human | Experimentally Observed,Moderate (predicted) | CRIM1 |
| rno-miR-20a-5p | TargetScan Human,miRecords | Experimentally Observed,High (predicted) | CXCL8 |
| rno-miR-20a-5p | Ingenuity Expert Findings,TarBase,TargetScan Human,miRecords | Experimentally Observed,High (predicted) | E2F1 |
| rno-miR-20a-5p | Ingenuity Expert Findings,TargetScan Human | Experimentally Observed,Moderate (predicted) | E2F2 |
| rno-miR-20a-5p | Ingenuity Expert Findings | Experimentally Observed | E2F3 |
| rno-miR-20a-5p | Ingenuity Expert Findings,TargetScan Human | Experimentally Observed,Moderate (predicted) | EGR2 |
| rno-miR-20a-5p | TargetScan Human,miRecords | Experimentally Observed,Moderate (predicted) | ESR1 |
| rno-miR-20a-5p | Ingenuity Expert Findings,TargetScan Human | Experimentally Observed,High (predicted) | HBP1 |
| rno-miR-20a-5p | TarBase,miRecords | Experimentally Observed | HIPK3 |
| rno-miR-20a-5p | miRecords | Experimentally Observed | ITCH |
| rno-miR-20a-5p | TargetScan Human,miRecords | Experimentally Observed,Moderate (predicted) | JAK1 |
| rno-miR-20a-5p | TargetScan Human,miRecords | Experimentally Observed,Moderate (predicted) | MAP3K12 |
| rno-miR-20a-5p | miRecords | Experimentally Observed | MEF2D |
| rno-miR-20a-5p | Ingenuity Expert Findings | Experimentally Observed | MICA |
| rno-miR-20a-5p | Ingenuity Expert Findings,TargetScan Human | Experimentally Observed,Moderate (predicted) | MMP3 |
| rno-miR-20a-5p | TarBase,miRecords | Experimentally Observed | MYLIP |
| rno-miR-20a-5p | TarBase,TargetScan Human,miRecords | Experimentally Observed,Moderate (predicted) | NCOA3 |
| rno-miR-20a-5p | TargetScan Human,miRecords | Experimentally Observed,Moderate (predicted) | PAK5 |
| rno-miR-20a-5p | Ingenuity Expert Findings,TargetScan Human | Experimentally Observed,High (predicted) | PKD2 |
| rno-miR-20a-5p | Ingenuity Expert Findings | Experimentally Observed | PPARG |
| rno-miR-20a-5p | Ingenuity Expert Findings,TargetScan Human | Experimentally Observed,Moderate (predicted) | PTEN |
| rno-miR-20a-5p | Ingenuity Expert Findings | Experimentally Observed | PURA |
| rno-miR-20a-5p | Ingenuity Expert Findings,TarBase,miRecords | Experimentally Observed | RB1 |
| rno-miR-20a-5p | TarBase,TargetScan Human,miRecords | Experimentally Observed,Moderate (predicted) | RBL2 |
| rno-miR-20a-5p | Ingenuity Expert Findings,miRecords | Experimentally Observed | RUNX1 |
| rno-miR-20a-5p | TargetScan Human,miRecords | Experimentally Observed,Moderate (predicted) | S1PR1 |
| rno-miR-20a-5p | Ingenuity Expert Findings,TargetScan Human,miRecords | Experimentally Observed,High (predicted) | STAT3 |
| rno-miR-20a-5p | Ingenuity Expert Findings,TarBase,TargetScan Human | Experimentally Observed,High (predicted) | TGFBR2 |
| rno-miR-20a-5p | Ingenuity Expert Findings | Experimentally Observed | TLR7 |
| rno-miR-20a-5p | Ingenuity Expert Findings | Experimentally Observed | TNF |
| rno-miR-20a-5p | Ingenuity Expert Findings | Experimentally Observed | TP63 |
| rno-miR-20a-5p | miRecords | Experimentally Observed | TUSC2 |
| rno-miR-20a-5p | miRecords | Experimentally Observed | VEGFA |
| rno-miR-20a-5p | miRecords | Experimentally Observed | VIM |
| rno-miR-20a-5p | TargetScan Human,miRecords | Experimentally Observed,High (predicted) | ZBTB7A |
